# Antibiotic Susceptibility of Environmental *Legionella pneumophila* Strains Isolated in Northern Italy

**DOI:** 10.3390/ijerph18179352

**Published:** 2021-09-04

**Authors:** Clementina Elvezia Cocuzza, Marianna Martinelli, Federica Perdoni, Chiara Giubbi, Maria Erica Alessandra Vinetti, Enrico Calaresu, Sergio Frugoni, Maria Scaturro, Maria Luisa Ricci, Rosario Musumeci

**Affiliations:** 1 Department of Medicine and Surgery, University of Milano-Bicocca, 20900 Monza, Italy; marianna.martinelli@unimib.it (M.M.); federica.perdoni@unimib.it (F.P.); chiara.giubbi@unimib.it (C.G.); ericavinetti@gmail.com (M.E.A.V.); e.calaresu1@gmail.com (E.C.); sergio.frugoni@gmail.com (S.F.); rosario.musumeci@unimib.it (R.M.); 2 MicroMiB Biorepository, University of Milano-Bicocca, Associated Member of the JRU MIRRI-IT, Via Cadore, 20900 Monza, Italy; 3 National Reference Laboratory for Legionella, Department of Infectious Diseases, Istituto Superiore di Sanità, 00161 Roma, Italy; maria.scaturro@iss.it (M.S.); marialuisa.ricci@iss.it (M.L.R.)

**Keywords:** *Legionella pneumophila*, antimicrobial susceptibility testing, minimum inhibitory concentrations, macrolides, fluoroquinolones, rifampicin, doxycycline, LpeAB active efflux system, reserpine coupled REMA assay

## Abstract

*Legionella pneumophila* is ubiquitous in aquatic environments and responsible for severe pneumonia in humans through inhalation of aerosol containing *Legionella* spp. Macrolides and fluoroquinolones are frequently used antimicrobials, but treatment failures are increasingly being reported. As susceptibility testing is not routinely performed, this study aimed to determine the minimum inhibitory concentrations (MICs) on 58 environmental *Legionella pneumophila* strains (24 of serogroup 1 and 34 of non-serogroup 1) isolated in Northern Italy. MICs of azithromycin, erythromycin, ciprofloxacin, levofloxacin, and rifampicin were determined by the microdilution method using buffered yeast extract broth supplemented with α-ketoglutarate (BYEα). Seventy-five percent of *Legionella pneumophila* isolates showed MIC values below the tentative highest MICs indicated by the European Committee on Antimicrobial Susceptibility Testing (EUCAST); rifampicin was the most active agent with MIC_90_ values below 0.008 mg/L. Interestingly, one isolate was tested and found to be PCR-positive for the azithromycin LpeAB active efflux system, further confirmed by the reserpine/resazurin microtiter assay. In conclusion, this study has provided additional susceptibility data for environmental *Legionella pneumophila* isolates from Northern Italy demonstrating, in general, low MICs values for the tested antimicrobials, although one strain tested was shown to possess the LpeAB resistance determinant, indicating that future surveillance studies are warranted.

## 1. Introduction

*Legionella* species (spp.) are aerobic, non-spore forming Gram-negative bacteria, which are ubiquitous in freshwater habitats, such as rivers, lakes, ponds, and hot springs, as well as colonizing artificial aquatic environments. In these environments, *Legionella* are naturally part of microbial ecosystems, found to be associated with complex biofilm communities as well as able to infect and replicate inside eukaryotic hosts, such as free-living amoebae, conferring them resistance and protection [1,2,3,4]. Through their ability to thrive within both amoebae and biofilm, *Legionellae* can enter and colonize man-made aquatic environments, from the initial point of water treatment, to tap water in private homes, hospitals, hotels, and, eventually, waste-water facilities [1]. Inhalation or aspiration of aerosol containing *Legionella pneumophila*, such as those that can be generated by showers, air-conditioning cooling towers, whirlpool spas and fountains, can result in human disease.

*Legionella pneumophila* (*Lp*) is most frequently involved in human infections, which can present as distinct clinical manifestations of disease —Legionnaires’ disease (LD), a serious pneumonia that can result in permanent lung damage or death, and Pontiac fever, a milder influenza-like disease as well as rarer forms of extra pulmonary infection [5,6,7].

The ability of *Lp* to grow intracellularly within pulmonary macrophages is a prerequisite for the development of LD. For this reason, antimicrobials able to achieve appropriate therapeutic concentrations within eukaryotic cells, such as erythromycin, rifampicin, tetracyclines, and fluoroquinolones, have been most efficacious in the treatment of Legionnaires’ disease, with azithromycin or levofloxacin generally being the antibiotics of choice. [8] Treatment is, however, often empiric, as laboratories do not routinely perform antibiotic susceptibility testing on *Legionella* isolates, due not only to the difficulty in isolating this pathogen from clinical samples, but also to the lack of specific standardized antibiotic susceptibility testing (AST) methods [9]. No formal consensus international guidelines, such as those generally provided by the Clinical and Laboratory Standards Institute (CLSI) and the European Committee on Antimicrobial Susceptibility Testing (EUCAST), are presently available for antibiotic susceptibility determination of *Lp.* As a result, there are also no ‘epidemiological cut-off’ (ECOFF) values for *Legionella* spp., defined as the in vitro MIC threshold that enables the discrimination of wild-type (WT) strains from those with acquired resistance mechanisms [9].

Despite rare reports of acquired antimicrobial resistance in *Legionella* [10,11,12,13,14,15,16,17,18], treatment failures have been reported following appropriate clinical use of macrolides and/or fluoroquinolones in the management of patients with LD [19,20,21,22,23,24,25]. Antibiotic resistance to ciprofloxacin has been reported in *Lp* resulting from a single point mutation in the *gyr*A gene [26], as well as reduced susceptibility to azithromycin (MICs 0.125–2 mg/L) associated to the presence of an LpeAB efflux pump system, a homologous system to *Escherichia coli* AcrAB-TolC, representing a tripartite efflux pump of the resistance–nodulation–division (RND) family [10,11,27,28].

Development of antibiotic resistance by bacterial pathogens represents a major public health concern worldwide, reducing the effectiveness of antibacterial treatment in clinical practice; it has been estimated that nosocomial infections caused by multi-resistant bacteria cause about 33,110 deaths per year in Europe [29]. The selection of antibiotic-resistant bacteria is not only related to the clinical setting but also to the natural environment, understood as the habitat and biological conditions in which an organism lives, which seems to have a fundamental role in the development and spreading of antibiotic resistance [30,31,32].

Water systems represent an important reservoir for the transmission of *Lp* infections, but in spite of this, antimicrobial susceptibility of wild-type isolates is usually not performed. The aim of this study was to evaluate susceptibility of environmental strains of *Legionella pneumophila* isolated in building water systems of some provinces of Northern Italy to antimicrobial agents commonly used in the therapy of LD, in order to acquire additional data on *Lp* susceptibility in this geographical area as well as allowing to foresee the potential emergence of antibiotic resistance in clinical isolates.

*Legionella pneumophila*, according to Italian legislation, represents a great concern not only in hospital and health care facilities hosting people with increased susceptibility to the disease are present, but also in workplaces and accommodation sites [33,34,35,36].

## 2. Materials and Methods 

### 2.1. Bacterial Strains

A total of 58 environmental strains of *Legionella pneumophila* were analyzed in this study. All strains were isolated between October 2018 and March 2019 from building water systems, including hotels, residential health-care structures, and office buildings situated in the Lombardy provinces of Monza-Brianza, Varese, Milan, and Como in Northern Italy.

All strains isolated and identified during the present study, characterized for antibiotic resistance profiles, are stored at −80 °C as part of the MicroMiB biorepository, associated member of the Joint Research Unit (JRU) MIRRI-IT (Microbial Resource Research Infrastructure—Italian Node), located at the University of Milano-Bicocca, Monza, Italy, for further characterization studies [37].

All isolates were identified and typed by Microgen^®^ kit (Microgen Bioproducts, Camberley, UK) latex test. According to the results, 24 isolates belonged to the Lp1 (“Lp1”) and 34 strains to the Lp 2–15 (“Lp2–15”) serogroups.

*Legionella pneumophila* NCTC 12821 (Vitroids^™^ Sigma^®^, Sigma-Aldrich, St. Louis, MO, USA) and *Staphylococcus aureus* ATCC 29213 (KWIK-STIK^™^ Microbiologics^®^, St. Cloud, MN, USA) were used as control organisms for susceptibility testing.

### 2.2. Antimicrobial Agents

Standard laboratory powders of the following antimicrobial agents were tested against bacterial strains: azithromycin, erythromycin, ciprofloxacin, levofloxacin, rifampicin, doxycycline, and tigecycline (tetracyclines were tested on a reduced number of environmental strains, 35 and 11 for doxycycline and tigecycline respectively) (Sigma-Aldrich).

Stock solutions were prepared fresh and further diluted in buffered yeast extract broth supplemented with α-ketoglutarate (BYEα) (BioLife Italiana, Milan, Italy) to obtain antibiotic concentrations following serial 2-fold dilutions starting from 32 µg/mL up to 0.016 mg/L.

### 2.3. Minimum Inhibitory Concentrations (MICs) Determination 

MIC determination was performed by broth microdilution (BMD) method, as previously described [9,10,38,39]. Briefly, *Lp* strains were inoculated on Buffered Charcoal Yeast Extract (BCYE) agar (Oxoid, Milan, Italy) and incubated for 72 h at 37 °C in a humified atmosphere (50% relative humidity). Isolated colonies were resuspended in 5 mL of buffered yeast extract broth supplemented with α-ketoglutarate (BYEα) (BioLife Italiana, Milan, Italy) and further incubated for 48 h at 37 °C in a humidified atmosphere. Broth cultures were then adjusted to a turbidity of 0.5 McFarland standard using fresh BYEα broth and further diluted 1:100 to give a bacterial concentration of approximately 1 × 10^6^ CFU/mL.

A 50 µL aliquot of each bacterial suspension was then dispensed into 96-well microtiter plates containing an equal volume of broth containing 2-fold serial dilutions of the test antimicrobial agents, giving a final bacterial concentration of 5 × 10^5^ CFU/mL in a total volume of 100 µL. The microtiter plates were incubated for 72 h at 37 °C in 5% CO_2_ in a humidified thermostat. The MIC was defined as the drug concentration in the first well with no visible growth.

MIC_50_ and MIC_90_ values were defined as the lowest concentration of the antimicrobial agent at which 50 and 90% respectively of the isolates were inhibited.

*Legionella pneumophila* NCTC 12821 and *Staphylococcus aureus* ATCC 29213 were used as control organisms for susceptibility testing, performed using BYEα and Muller–Hinton (MH) broths.

### 2.4. PCR Amplification of LpeAB Gene

The presence of the *lpe*AB gene was evaluated by PCR amplification for one of the environmental Lp1 isolates (VA35, Table S1 in Supplementary Material) with azithromycin MIC value of 1 mg/L and for one of the Lp2–15 isolates with MIC of 8 mg/L (VA31). The *lpe*AB gene PCR assay was performed according to protocol previously described by Vandewalle-Capo et al. [11]. Three clinical isolates (OSP1, OSP2, and OSP3, Table S1 in Supplementary Material) belonging to Lp1 and with azithromycin MIC values of 1 mg/L were also included in the screening assay. An Lp2–15 environmental isolate (VA18) with MIC ≤ 0.008 mg/L for azithromycin and an Lp1 clinical isolate (OSP4) with MIC = 0.12 mg/L were also used as control strains with lower MIC values.

### 2.5. Inhibition of LpeAB Efflux Pump Activity by Reserpine/Resazurin Microtiter Assay

To demonstrate the quantitative inhibition of the LpeAB efflux pump activity, reserpine, an efflux pump inhibitor (EPI) of RND efflux pump systems, was added to the liquid media prior to azithromycin BMD MIC determination against *lpe*AB PCR-positive *Lp* strains (VA35, OSP1, OSP2, and OSP3) as well as two *lpe*AB PCR-negative isolates (VA31 and OSP4). Briefly, a fresh solution of reserpine (Sigma-Aldrich) was prepared dissolving 20 mg in 100 mL of DMSO. Similarly, 2 mg of resazurin (Sigma-Aldrich) was dissolved in distilled water (100 mL) to obtain a 0.02 wt% solution. BMD MIC determination for azithromycin was performed using two separate rows of the 96 wells microtiter plate as previously described; serial dilutions of azithromycin alone were included in the first row, whilst 10 μL of reserpine solution were added to the second row together with azithromycin serial dilutions. After incubation for 72 h at 37 °C in 5% CO_2_ in a humidified thermostat, 30 μL of resazurin solution was added to each well, adapting resazurin microtiter assay (REMA) in the presence or absence of reserpine EPI, as previously described [40]. Plates were read after a 24 h incubation at 37 °C; a change in color from blue to pink indicated the growth of bacteria, and the MIC was defined as the lowest drug concentration that prevented this change in color. Final azithromycin MIC values ranged from 8 to 0.004 mg/L.

## 3. Results

The 58 environmental *Lp* isolates used for antimicrobial susceptibility determination were further divided into 24 isolates belonging to serogroup 1 (Lp1) and 34 to serogroups 2 to 15 (Lp2–15).

The overall cumulative percentages of isolates inhibited by different concentrations of the 5 antimicrobial agents tested are shown in Table 1. Doxycycline and tigecycline were also tested but on a reduced number of strains, 35 and 11 environmental strains, respectively; these results are shown in Table S1 in Supplementary Material. Although insufficient data is presently available to establish epidemiological cut off values (ECOFFs), tentative highest MIC values for the most commonly used antimicrobial agents against wild-type *Lp* have been published by EUCAST, dividing these values between Lp1 and Lp2–15 isolates [10,15,16].

Overall MIC_50_ and the MIC_90_ values are shown in Table 2. Rifampicin was found to be the most active agent with MIC_50_ and MIC_90_ lower than 0.008 mg/L.

The MIC_50_ and the MIC_90_ values for *Legionella pneumophila* isolates divided according to Lp1 and Lp2–15 isolates are shown in Table 3.

Rifampicin was shown to be the most active drug with MIC_50_ and MIC_90_ lower than 0.008 mg/L for both Lp1 and Lp2–15 strains, with MIC ranges of ≤0.008 mg/L and ≤0.008–0.015 mg/L, respectively. All *Lp* isolates except one (MIC = 0.015 mg/L) showed MICs below the highest tentative MIC concentrations indicated by EUCAST for rifampicin (<0.002 mg/L for Lp1-belonging and 0.008 mg/L for Lp2–15-belonging strains) [10].

Azithromycin and erythromycin showed higher MIC_50_ and MIC_90_ values against Lp1 isolates as compared to Lp2–15 strains, whilst MIC_50_ and MIC_90_ for ciprofloxacin and levofloxacin showed similar values for both Lp1 and Lp2–15 isolates. 

Complete MIC value results for the tested antimicrobial agents against all tested *Lp* isolates are shown in Table S1 in Supplementary Material.

Interestingly, only one Lp2–15 strain out of all tested *Lp* isolates (1/58, 1.7%) showed MIC values of 8 mg/L for both azithromycin and erythromycin, higher than the tentative highest MIC concentrations indicated by EUCAST for azithromycin (0.125 mg/L for Lp1 LpeAB-negative and for WT *Lp* and 2 mg/L for Lp1 LpeAB-positive strains) and erythromycin (1 mg/L) [10]. The highest MIC values for ciprofloxacin and levofloxacin (0.5 mg/L) were also demonstrated for the same isolate.

Another Lp2–15 strain (MB12 in Table S1) showed MICs of 1 mg/L and 0.25 mg/L for azithromycin and erythromycin, respectively, while three Lp1 strains (MB1, VA34, and VA35) showed MICs of 0.5–1 and 0.5 mg/L for the two tested macrolides, respectively. 

In this study, *Lp* isolates with MIC values above the tentative highest MIC for wild-type organisms, as indicated by EUCAST [10], were shown to be 6.9% (4/58) for azithromycin and 1.7% (1/58) for erythromycin.

For fluoroquinolones, the highest tentative MIC concentrations indicated by EUCAST were 0.03 mg/L for ciprofloxacin for all the serogroups, 0.03 mg/L (Lp1) and 0.125 mg/L (Lp2-15) for levofloxacin [10]. The studied environmental strains showed rates of 15.5% and 1.7% of “not wild-type” isolates against ciprofloxacin and levofloxacin, respectively. Interestingly, all of these “not wild-type” strains belonged to the Lp2–15 group.

For doxycycline, only 35/58 (60.3%) of the environmental strains under investigation were tested which showed the highest MICs distribution (MIC_Range_ = 0.25–8 mg/L, MIC_50_ = 4 mg/L, MIC_90_ = 8 mg/L) with 6/35 (17.1%) strains found to belong to the “not wild-type”, according to EUCAST guidelines. Interestingly, none of 23/35 (65.7%) strains belonging to the Lp2–15 group presented a “not wild-type” phenotype. 

In this study, the presence of the *lpe*AB gene by PCR amplification of a 359 bp product was demonstrated in the VA35 Lp1 environmental strain, with MIC = 1 mg/L for azithromycin and 0.5 mg/L for erythromycin, as well as in three clinical strains (OSP1–OSP3) of *Lp* belonging to the serogroup 1, as shown in Figure S1 in Supplementary Material. On the contrary, in the VA31 Lp2–15 strain, showing azithromycin MIC values of 8 mg/L, which are higher than those previously reported [11] as associated to the presence of LpeAB efflux pump system (MICs 0.125–2 mg/L), the *lpe*AB gene was not demonstrated.

To confirm the effect of the LpeAB system on active azithromycin efflux, a reserpine–REMA assay was combined to MIC determination for azithromycin by the BMD method in BYEα medium against the four *Lp* strains demonstrating *lpe*AB presence by PCR amplification (VA35, OSP1–OSP3). As shown in Figure S2, the effect of LpeAB efflux pump inhibition by reserpine was demonstrated by a 16 to 32-fold decrease of azithromycin MICs in these isolates. For OSP1, OSP3, and VA35 strains, a 16-fold decrease of MIC of azithromycin was observed, associated with an MIC shift from 1 mg/L to 0.06 mg/L. For OSP2, MIC values changed from 1 mg/L to 0.03 mg/L. For OSP4, the *lpe*AB PCR-negative isolate, no effect was observed following exposure to reserpine, confirming MIC values of 0.12 mg/L. Finally, also in the case of the VA31 strain with a MIC of azithromycin values of 8 mg/L and a negative amplification of *lpe*AB gene, no effect of reserpine was observed on MIC determination.

## 4. Discussion

*Legionella pneumophila* is a widespread and ubiquitous microorganism in water systems from which it can be acquired and became potentially pathogenic to man. Environmental surveillance of *L. pneumophila* with reduced antibiotic susceptibility can be important in order to predict the evolution of antibiotic resistance, which can have an impact on the antibiotic treatment for the clinical management of LD [11].

Sanitary systems of various structures, such as outpatient clinics, residences for the elderly, sports centers, hotels, condominiums, were checked for the presence of *Lp* and two main serogroups, Lp1 and Lp2–15, were identified.

Despite the severity of human disease, treatment choice is generally empiric as there are, to date, few of reports on the antimicrobial susceptibility of environmental *L. pneumophila* [11].

The fastidious nature of *Lp* results in the difficulty not only in isolating this pathogen from clinical samples and environmental matrixes, such as amoebae and complex biofilms, but also in performing antimicrobial susceptibility testing. Moreover, the lack of standardized methods for *L. pneumophila* susceptibility testing has resulted in the failure to establish appropriate clinical MIC cut-off values, allowing to correlate in vitro results to clinical outcome. There is, therefore, presently a great quest among researchers, stakeholders, and reference laboratories for the standardization of antimicrobial susceptibility testing methods, as well as for new guidelines and reference strains to facilitate and improve the detection of antimicrobial resistance [41].

In this study, the susceptibility against one second line and five first line antimicrobial agents was evaluated in 58 environmental strains belonging to both serogroup 1 (24) and serogroups 2–15 (34). A broth microdilution method based on BYEα medium was used in this study in order to better standardize *Lp* antimicrobial susceptibility testing which, although more time-consuming than gradient tests, provides unbiased MICs results due to the absence of charcoal [10].

Due to the lack of standardization and to the wide range of methods used for antimicrobial susceptibility testing of *Lp*, the comparison of MICs values described in different studies can be controversial [11,12,13,14,15,16,17,18,19,20]. The results of the present investigation were therefore compared with those of recent reports in which a MDB method was used to evaluate *Lp* susceptibility [9,11,12]. A perfect overlap was observed in *Lp* susceptibility to rifampicin when comparing results with those of these previous reports, with MIC_50_ and MIC_90_ values ≤ 0.008 mg/L [9,11,12]. Susceptibility to ciprofloxacin was also shown to be in keeping with that demonstrated in two of the previous studies in which this antimicrobial agent was evaluated, with only slightly higher MIC_90_ values (MIC_90_ = 0.06 mg/L compared to the previously reported value of 0.03 mg/L) [11,12]. For levofloxacin, MIC_50_ and MIC_90_ values were also overall concordant with those of previous studies, although Wilson et al. study reported higher MIC_50_ and MIC_90_ values (0.06 mg/L and 0.125 mg/L respectively). Furthermore, lower MIC values for levofloxacin (MIC_50_ ≤ 0.008 mg/L and MIC_90_ = 0.015 mg/L) were generally observed in this study for Lp 2–15 isolates as compared to Lp 1. Azithromycin MIC_50_ = 0.06 mg/L and MIC_90_ = 0.5 mg/L values reported in the present study were identical to those described by Vandewalle-Capo et al. for the Lp1 group and comparable to those observed by Portal et al. (MIC_50_ = 0.03 mg/L and MIC_90_ = 0.06 mg/L)); higher MIC values for azithromycin were found to be associated with the Lp2–15 isolates analyzed in our study (MIC_50_ = 0.015 mg/L and MIC_90_ = 0.03 mg/L). For erythromycin, a relatively good concordance between MIC_50_ and MIC_90_ values for the studied Lp1 isolates (MIC_50_ = 0.12 mg/L and MIC_90_ = 0.5 mg/L) and those reported by both Vandewalle et al. (MIC_50_ = 0.12 mg/L and MIC_90_ = 0.5 mg/L) and Wilson et al. (MIC_50_ = 0.25 mg/L and MIC_90_ = 0.5 mg/L) was observed. For Lp2–15 lower MIC_50_ (0.06 mg/L) and MIC_90_ (0.12 mg/L) values were shown for the strains under investigation. Finally in the present study, isolates showed MIC_50_ = 4 mg/L and MIC_90_ = 8 mg/L for doxycycline, which were found to be lower than those reported by Portal et al. (MIC_50_ = 16 mg/L and MIC_90_ = 32 mg/L) but higher than those described by Vandewalle-Capo et al. (MIC_50_ = 1 mg/L and MIC_90_ = 2 mg/L).

Comparable MIC distributions and results achieved in studies using a common methodology, further support the indications by Portal et al. who advocate the use of the BMD method in combination with BYEα medium for AST of *Lp* [41].

Furthermore, the majority of the investigated wild-type isolates analyzed in this study demonstrated MIC values overlapping with the tentative wild-type distribution suggested by EUCAST and others [9,10,15,16], although a few strains were shown to have MICs above the tentative highest MIC values for wild-type organisms. Reduced susceptibility to ciprofloxacin was observed in the present study in 9/58 (15.5%) of tested strains, all belonging to the Lp2–15 group (MIC values above the 0.03 mg/L EUCAST tentative highest MIC values); only one of these isolates demonstrated the same phenotype for levofloxacin. Moreover, for azithromycin two of the environmental Lp2–15 strains were also shown to have MICs above EUCAST tentative wild-type distribution (MIC of 1 and 8 mg/L, respectively). The presence of the LpeAB efflux pump system was confirmed in one of the studied Lp1 environmental strains showing MIC of 1 mg/L for azithromycin but interestingly not in the Lp2–15 isolate with MIC of 8 mg/L. Isolates with elevated azithromycin MICs (MIC = 0.5–1 mg/L) were found to have erythromycin MICs below EUCAST tentative highest MICs, with the exception of the Lp2–15 isolate negative for the LpeAB efflux pump system and showing MICs of 8 mg/L for both azithromycin and erythromycin. In keeping with the data reported by Natås et al, MICs values of erythromycin do not allow to unveil the presence of an LpeAB efflux system as part of antibiotic susceptibility testing [14]. For rifampicin only one strain demonstrated a MIC value of 0.015 mg/L, above EUCAST tentative highest wild-type MIC. For doxycycline 6/12 Lp1 isolates showed MICs above tentative highest wild-type MIC = 2 mg/L, whilst all 23 strains belonging to Lp2–15 demonstrated MICs below the tentative highest wild-type MIC equal to 32 mg/L. Considering the overall distribution of doxycycline MICs observed in this study, with the highest documented MIC values of 8 mg/L for both Lp1 and Lp2–15 isolates, in our opinion, the tentative highest wild-type MIC value of 32 mg/L for the Lp2–15 group may need to be reconsidered based on the evaluation of a higher number of isolates.

As there are presently no defined clinical breakpoints for *Legionella*, clinical isolates with reduced susceptibility may represent a potential risk for patient treatment, and further surveillance studies are warranted.

## 5. Conclusions

In this study we analyzed 58 recently isolated environmental *Legionella pneumophila* strains taking also into account their serogroup (*Lp*1 or *Lp*2–15) and their antibiotic susceptibility phenotypes, by means of a BMD method according to the recently revised EUCAST Guidance document on Legionella susceptibility testing (May 2021). This guidance document suggests that the MBD method provides unbiased MICs results due to the absence of charcoal as well as allowing to report MIC distributions for the most commonly used antibacterial agents and tentative highest MICs for the wild-type population.

Out of the 58 *Lp* isolates evaluated in the present study, 12 (20.7%) demonstrated “not wild-type” susceptibility phenotypes against one or more of the 5 different antibiotics belonging to 3 different classes. Also considering doxycycline, which was tested on a reduced number of strains, the overall rate of “not wild-type” strains increased to 31%. Of note, 50% (6/12) of Lp1 strains demonstrating reduced susceptibility to doxycycline were found to be susceptible to all the other tested antibiotics, suggesting a cluster of *L. pneumophila* strains with specific resistance to an antibiotic representing a second-line therapeutic option for the treatment of mild severity LD in immunocompetent subjects, according to the Italian guidelines for the prevention and treatment of legionellosis [25].

To the best of our knowledge, this is the first study demonstrating the role of the lpeAB efflux pump system in isolates with reduced susceptibility to azithromycin, by evaluating the shift in MIC values in the presence and absence of the efflux pump inhibitor reserpine. 

In the future, further typing of *Lp* isolates showing elevated MIC values would allow to establish the clonal relationship of isolates with reduced antimicrobial susceptibility and to compare these clonal types with those associated with human disease.

## Figures and Tables

**Table 1:** Cumulative MICs (mg/L) distribution of 58 environmental isolates of *Legionella pneumophila*.

Antimicrobial Agents	Cumulative Percentage (%) of Strains Inhibited at Indicated Antimicrobials Concentrations (mg/L)											
	≤0.008	0.015	0.03	0.06	0.12	0.25	0.5	1	2	4	8	16
**Azithromycin**	1.7	39.6	72.4	84.4	91.3		93.1	98.3			100	
**Erythromycin**		5.2	19	63.8	89.6	93.1	98.3				100	
**Ciprofloxacin**	8.6	52.2	84.5	94.8	98.3		100					
**Levofloxacin**	37.9	84.5	96.5		98.3		100					
**Rifampicin**	98.3	100										

**Table 2:** MIC_Range_, MIC_50_, and MIC_90_ values (mg/L) for 58 environmental isolates of *Legionella pneumophila*.

Antimicrobial Agents	MIC_50_	MIC_90_	MIC_Range_
**Azithromycin**	0.03	0.12	≤0.008–8
**Erythromycin**	0.06	0.12	0.015–8
**Ciprofloxacin**	0.015	0.06	≤0.008–0.5
**Levofloxacin**	0.015	0.03	≤0.008–0.5
**Rifampicin**	≤0.008	≤0.008	≤0.008–0.15

**Table 3:** MIC_Range_, MIC_50_, and MIC_90_ values (mg/L) of *Legionella pneumophila* serogroup 1 (Lp1) isolates compared with serogroup 2–15 (Lp2–15).

Bacterial Strains (n° of Isolates)	Antimicrobial Agents	MIC_50_	MIC_90_	MIC_Range_
***L. pneumophila* Lp1** ***(24)***	**Azithromycin**	0.06	0.5	0.015–1
	**Erythromycin**	0.12	0.5	0.015–0.5
	**Ciprofloxacin**	0.015	0.03	≤0.008–0.03
	**Levofloxacin**	0.015	0.03	≤0.008–0.03
	**Rifampicin**	≤0.008	≤0.008	≤0.008
***L. pneumophila* Lp2–15** ***(34)***	**Azithromycin**	0.015	0.03	≤0.008–8
	**Erythromycin**	0.06	0.12	0.015–8
	**Ciprofloxacin**	0.015	0.06	≤0.008–0.5
	**Levofloxacin**	≤0.008	0.015	≤0.008–0.5
	**Rifampicin**	≤0.008	≤ 0.008	≤0.008–0.015

## Supplementary Materials

The following are available online at https://www.mdpi.com/article/10.3390/ijerph18179352/s1, Table S1: Characteristics of 58 environmental and 4 clinical isolates of *Legionella pneumophila* and antibiotic susceptibility patterns; Figure S1: 2% Agarose gel electrophoresis of amplified *lpe*AB PCR products (359 bp) obtained from three clinical (OSP1–3) and one (VA35) environmental *L. pneumophila* isolates, Figure S2: Reserpine–resazurin assay for confirmation of LpeAB efflux system in environmental *L. pneumophila* isolates.
