# Supplementary material for: Antibiotic Susceptibility of Environmental Legionella pneumophila Strains Isolated in Northern Italy
Source: Int J Environ Res Public Health. 2021 Sep 4;18(17):9352. doi: 10.3390/ijerph18179352 (PMC8431511; doi:10.3390/ijerph18179352)
Supplement: Supplementary file 1 [file ijerph-18-09352-s001.zip › Figure S2 - Reserpine-resazurin Assay for confirmation of LpeAB efflux system.pdf]

A

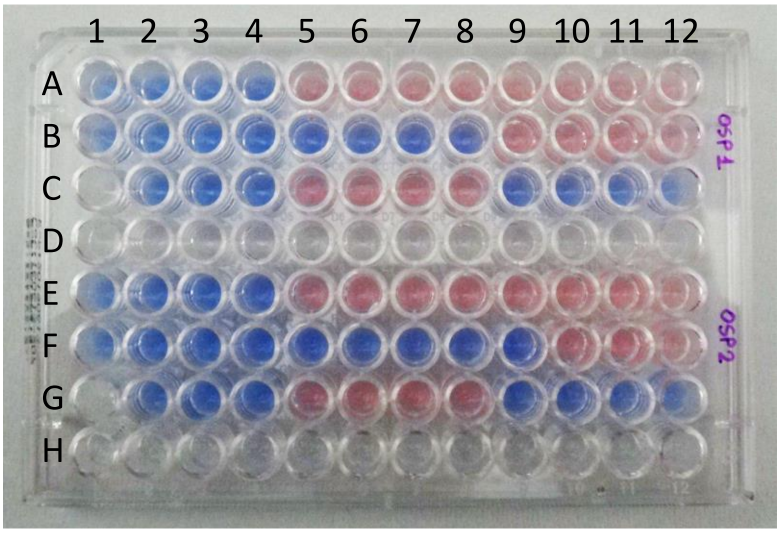

OSP1 (*lpe*AB +ve) AZT MIC = 1 mg/L

+ reserpine

OSP2 (*lpe*AB +ve) AZT MIC = 1 mg/L

+ reserpine

B

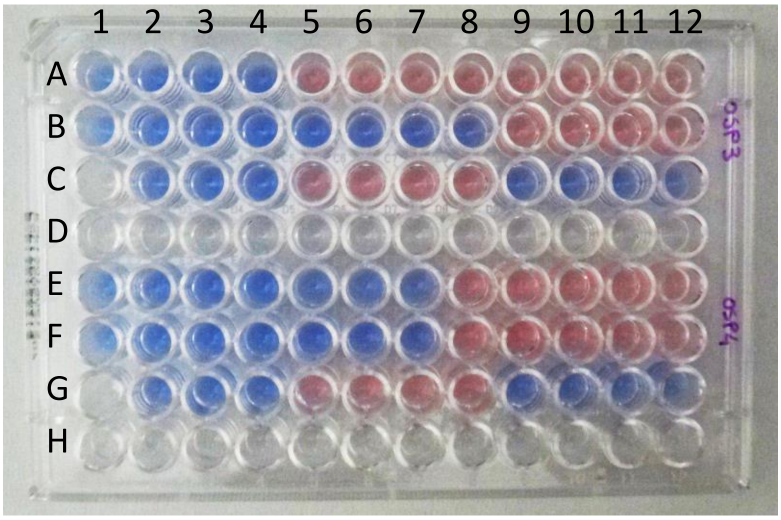

OSP3 (*lpe*AB +ve) AZT MIC = 1 mg/L

+ reserpine

OSP4 (*lpe*AB -ve) AZT MIC = 0.12 mg/L

+ reserpine

C

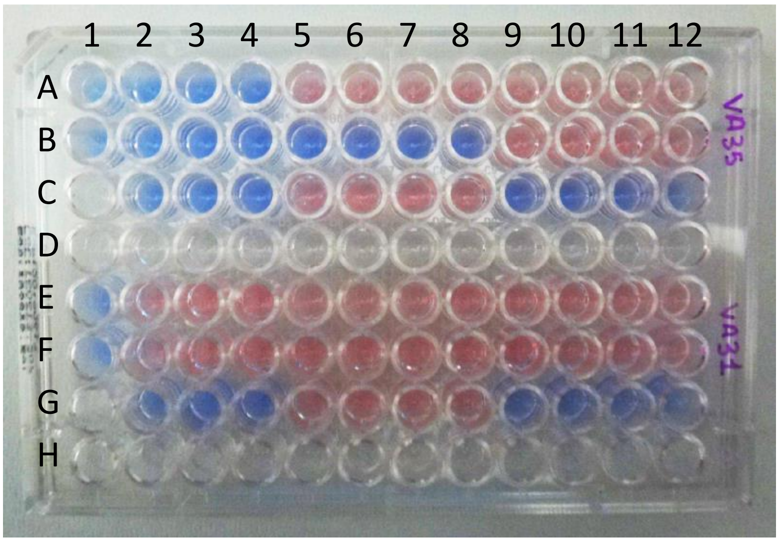

VA35 (*lpe*AB +ve) AZT MIC = 1 mg/L

+ reserpine

VA31 (*lpe*AB -ve) AZT MIC = 8 mg/L

+ reserpine

Figure S2 - Reserpine-resazurin Assay for confirmation of LpeAB efflux system in environmental *L. pneumophila* isolates. In each plate 2 different strains are evaluated. Rows A-E = MIC determination of azithromycin well concentrations ranging from 8 to 0.004 mg/L. Rows B-F = MIC determination of azithromycin concentrations ranging from 8 to 0.004 mg/L plus reserpine. Rows C-G = negative controls (only BYEα medium, wells 2-4), positive controls (BYEα medium and bacterial inoculum, wells 5-8), reserpine control (BYEα medium plus reserpine, wells 9-12), C1-G1 and Rows D-H = empty wells. By using resazurin microtiter assay, change in color from blue to pink indicated change of bacterial viability. MIC (mg/L) was defined as the lowest concentration of drug that prevented this change in color.
