# Supplementary material for: Antibiotic Susceptibility of Environmental Legionella pneumophila Strains Isolated in Northern Italy
Source: Int J Environ Res Public Health. 2021 Sep 4;18(17):9352. doi: 10.3390/ijerph18179352 (PMC8431511; doi:10.3390/ijerph18179352)
Supplement: Supplementary file 1 [file ijerph-18-09352-s001.zip › Figure S1 - Agarose Gel electrophoresis for lpeAB component.pdf]

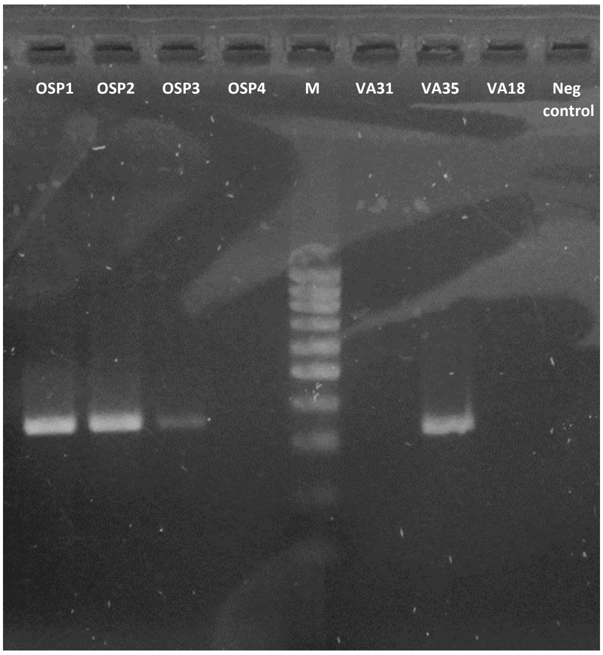

Figure S1: 2% Agarose gel electrophoresis of amplified *lpe*AB PCR products (359 bp) obtained from 3 clinical (OSP1-3) and 1 (VA35) environmental *L. pneumophila* isolates.

M = 100bp ladder (Promega Corporation)
