# Supplementary material for: Antibiotic Susceptibility of Environmental Legionella pneumophila Strains Isolated in Northern Italy
Source: Int J Environ Res Public Health. 2021 Sep 4;18(17):9352. doi: 10.3390/ijerph18179352 (PMC8431511; doi:10.3390/ijerph18179352)
Supplement: Supplementary file 1 [file ijerph-18-09352-s001.zip › Table S1 - Characteristics of tested Legionella pneumophila strains and MIC values.pdf]

			Antimicrobial compounds and MIC values (mg/L)						
**Sample**	**Source**	**Serogroups**	**AZT**	**E**	**CIP**	**LEV**	**RF**	**DOX***	**TIG****
			**Environmental *Legionella pneumophila* strains**						
**MB1**	Outpatient Clinics	1	1	0,5	0,015	0,015	≤ 0,008	ND	ND
**MB2**	Outpatient Clinics	2 – 15	0,03	0,12	0,03	0,015	≤ 0,008	ND	ND
**MB3**	Outpatient Clinics	1	0,06	0,06	0,03	0,015	≤ 0,008	ND	ND
**MB4**	Outpatient Clinics	1	0,12	0,12	0,03	0,03	≤ 0,008	ND	ND
**MB5**	Outpatient Clinics	1	0,12	0,12	0,03	0,03	≤ 0,008	ND	ND
**MB6**	Outpatient Clinics	1	0,06	0,12	0,015	0,015	≤ 0,008	ND	ND
**MB7**	Outpatient Clinics	1	0,06	0,12	0,03	0,015	≤ 0,008	ND	ND
**MB8**	Outpatient Clinics	1	0,06	0,12	0,03	0,015	≤ 0,008	ND	ND
**MB9**	Outpatient Clinics	1	0,03	0,06	0,03	0,03	≤ 0,008	ND	ND
**MB10**	Outpatient Clinics	1	0,06	0,12	0,03	0,03	≤ 0,008	ND	ND
**MB11**	Outpatient Clinics	1	0,12	0,12	0,03	0,015	≤ 0,008	ND	ND
**MB12**	Outpatient Clinics	2 – 15	1	0,25	≤ 0,008	≤ 0,008	≤ 0,008	ND	ND
**MB13**	Outpatient Clinics	1	0,03	0,12	0,03	0,015	≤ 0,008	ND	ND
**VA14**	Restaurant	1	0,03	0,03	0,015	≤ 0,008	≤ 0,008	ND	ND
**VA15**	Health Care Residence	2 – 15	0,015	0,06	0,015	≤ 0,008	≤ 0,008	ND	ND
**VA16**	Health Care Residence	2 – 15	0,015	0,06	0,015	≤ 0,008	≤ 0,008	ND	ND
**VA17**	Health Care Residence	2 – 15	0,015	0,06	0,015	≤ 0,008	≤ 0,008	ND	ND
**VA18**	Health Care Residence	2 – 15	≤ 0,008	0,06	0,015	≤ 0,008	≤ 0,008	ND	ND
**VA19**	Health Care Residence	2 – 15	0,015	0,03	0,015	≤ 0,008	≤ 0,008	ND	ND
**VA20**	Health Care Residence	2 – 15	0,015	0,06	≤ 0,008	≤ 0,008	≤ 0,008	ND	ND
**VA21**	Health Care Residence	2 – 15	0,015	0,06	0,03	≤ 0,008	≤ 0,008	ND	ND
**VA22**	Health Care Residence	2 – 15	0,015	0,06	0,015	0,015	≤ 0,008	ND	ND
**VA23**	Health Care Residence	2 – 15	0,015	0,06	0,015	≤ 0,008	≤ 0,008	ND	ND
**VA24**	Health Care Residence	2 – 15	0,015	0,06	0,03	0,015	≤ 0,008	4	ND
**VA25**	Health Care Residence	2 – 15	0,015	0,06	0,03	0,015	≤ 0,008	2	ND
**VA26**	Health Care Residence	2 – 15	0,015	0,12	0,015	0,015	≤ 0,008	1	ND
**VA27**	Sports Centre	2 – 15	0,03	0,03	≤ 0,008	≤ 0,008	≤ 0,008	8	ND
**VA28**	Health Care Residence	1	0,06	0,12	0,03	0,015	≤ 0,008	8	ND
**VA29**	Health Care Residence	2 – 15	0,03	0,06	0,015	0,015	≤ 0,008	0,5	16
**VA30**	Health Care Residence	2 – 15	0,03	0,06	0,015	0,015	≤ 0,008	0,5	16
**VA31**	Archiepiscopal Seminary	2 – 15	8	8	0,5	0,5	≤ 0,008	8	16
**VA32**	Chemical Factory	1	0,015	0,06	0,015	0,015	≤ 0,008	1	8
**VA33**	Chemical Factory	1	0,03	0,12	0,015	0,03	≤ 0,008	1	8
**VA34**	Research Centre	1	0,5	0,5	0,015	0,03	≤ 0,008	0,5	4
**VA35**	Research Centre	1	1	0,5	0,015	0,015	≤ 0,008	0,5	8
**VA36**	Research Centre	2 – 15	0,015	0,12	0,06	≤ 0,008	≤ 0,008	4	ND
**VA37**	Accomodation Site	2 – 15	0,015	0,03	0,12	≤ 0,008	≤ 0,008	8	ND
**VA38**	Accomodation Site	2 – 15	0,03	0,06	0,12	0,12	≤ 0,008	8	ND
**VA39**	Accomodation Site	2 – 15	0,03	0,12	0,03	≤ 0,008	≤ 0,008	4	ND
**MI40**	Health Care Residence	1	0,015	0,06	0,015	≤ 0,008	≤ 0,008	8	ND
**MI41**	Health Care Residence	2 – 15	0,015	0,015	≤ 0,008	≤ 0,008	≤ 0,008	8	ND
**MI42**	Accomodation Site	1	0,12	0,06	≤ 0,008	0,015	≤ 0,008	4	ND
**MI43**	Accomodation Site	1	0,03	0,12	0,015	≤ 0,008	≤ 0,008	8	ND
**MI44**	Health Care Residence	2 – 15	0,015	0,03	0,015	≤ 0,008	≤ 0,008	8	ND
**MI45**	Health Care Residence	2 – 15	0,015	0,03	0,015	≤ 0,008	≤ 0,008	8	ND
**MI46**	Health Care Residence	2 – 15	0,03	0,06	0,015	0,015	≤ 0,008	1	ND
**MI47**	Health Care Residence	2 – 15	0,03	0,06	0,015	0,015	≤ 0,008	0,5	8
**MI48**	Health Care Residence	2 – 15	0,03	0,06	0,03	0,03	≤ 0,008	0,25	4
**MI49**	Health Care Residence	2 – 15	0,03	0,03	0,015	0,015	≤ 0,008	0,5	4
**MI50**	Restaurant	1	0,03	0,06	0,03	0,015	≤ 0,008	0,5	2
**MI51**	Accomodation Site	1	0,015	0,015	0,015	0,015	≤ 0,008	0,5	ND
**MI52**	Pastoral Centre	2 – 15	0,03	0,06	0,06	0,015	≤ 0,008	8	ND
**MI53**	Pastoral Centre	2 – 15	0,03	0,06	0,06	0,015	≤ 0,008	8	ND
**MI54**	Pastoral Centre	2 – 15	0,03	0,06	0,06	0,015	≤ 0,008	8	ND
**MI55**	Hospital Ward	2 – 15	0,06	0,25	0,06	≤ 0,008	0,015	4	ND
**CO56**	Jointly-owned Block of Flats	1	0,015	0,03	0,03	≤ 0,008	≤ 0,008	4	ND
**CO57**	Jointly-owned Block of Flats	1	0,015	0,015	0,015	≤ 0,008	≤ 0,008	8	ND
**NO58**	Sanitization Company	2 – 15	0,015	0,06	0,06	0,015	≤ 0,008	2	ND
	**Clinical isolates of *Legionella pneumophila* from BAL**								
**OSP1**	Hospital Ward	1	1	0,5	0,015	0,015	≤ 0,008	1	16
**OSP2**	Hospital Ward	1	1	0,5	0,015	0,03	≤ 0,008	0,5	8
**OPS3**	Hospital Ward	1	1	0,5	0,03	0,03	≤ 0,008	0,5	16
**OSP4**	Hospital Ward	1	0,12	0,25	0,06	0,06	≤ 0,008	8	16

Table S1: Characteristics of 58 environmental and 4 clinical isolates of *Legionella pneumophila* and antibiotic susceptibility patterns

AZT=azithromycin, E=erythromycin, CIP=ciprofloxacin, LEV=levofloxacin, RF=rifampicin, DOX=doxycycline, TIG=tigecycline, ND=not determined

* doxycycline was tested against 35 environmental and 4 clinical *L. pneumophila* strains

** tigecycline was tested against 11 environmental and 4 clinical *L. pneumophila* strains
